# Superoxide signaling in perivascular adipose tissue promotes age-related artery stiffness

**DOI:** 10.1111/acel.12196

**Published:** 2014-01-21

**Authors:** Bradley S Fleenor, Jason S Eng, Amy L Sindler, Bryant T Pham, Jackson D Kloor, Douglas R Seals

**Affiliations:** 1 Department of Integrative Physiology, University of ColoradoBoulder, CO, USA; 2 Kinesiology and Health Promotion, Graduate Center for Nutritional Sciences, University of KentuckyLexington, KY, USA

**Keywords:** fat, oxidative stress, peri-aortic, TEMPOL

## Abstract

We tested the hypothesis that superoxide signaling within aortic perivascular adipose tissue (PVAT) contributes to large elastic artery stiffening in old mice. Young (4–6 months), old (26–28 months), and old treated with 4-Hydroxy-2,2,6,6-tetramethylpiperidine 1-oxyl (TEMPOL), a superoxide scavenger (1 mm in drinking water for 3 weeks), male C57BL6/N mice were studied. Compared with young, old had greater large artery stiffness assessed by aortic pulse wave velocity (aPWV, 436 ± 9 vs. 344 ± 5 cm s^-1^) and intrinsic mechanical testing (3821 ± 427 vs. 1925 ± 271 kPa) (both *P* < 0.05). TEMPOL treatment in old reversed both measures of arterial stiffness. Aortic PVAT superoxide production was greater in old (*P* < 0.05 vs. Y), which was normalized with TEMPOL. Compared with young, old controls had greater pro-inflammatory proteins in PVAT-conditioned media (*P* < 0.05). Young recipient mice transplanted with PVAT from old compared with young donors for 8 weeks had greater aPWV (409 ± 7 vs. 342 ± 8 cm s^-1^) and intrinsic mechanical properties (3197 ± 647 vs. 1889 ± 520 kPa) (both *P* < 0.05), which was abolished with TEMPOL supplementation in old donors. Tissue-cultured aortic segments from old in the presence of PVAT had greater mechanical stiffening compared with old cultured in the absence of PVAT and old with PVAT and TEMPOL (both, *P* < 0.05). In addition, PVAT-derived superoxide was associated with arterial wall hypertrophy and greater adventitial collagen I expression with aging that was attenuated by TEMPOL. Aging or TEMPOL treatment did not affect blood pressure. Our findings provide evidence for greater age-related superoxide production and pro-inflammatory proteins in PVAT, and directly link superoxide signaling in PVAT to large elastic artery stiffness.

## Introduction

Aging is the major risk factor for cardiovascular diseases (CVD), as nearly 90% of incident CV events occur in adults over 55 years of age (Go *et al*., 2013). Stiffening of the large elastic arteries (aorta and carotid arteries) is a strong, independent predictor of cardiovascular events with aging (Sutton-Tyrrell *et al*., 2005; Mitchell *et al*., 2010), and superoxide-dependent oxidative stress and inflammation are key mechanisms by which large elastic arteries stiffen with age (Kim *et al*., 2009; Sindler *et al*., 2011; Fleenor *et al*., 2012).

Perivascular adipose tissue (PVAT) surrounds large elastic arteries and may exert an important influence on arterial stiffness. Visceral white adipose tissues from older mice demonstrate greater oxidative stress, which may lead to greater pro-inflammatory cytokine and chemokine secretion (Findeisen *et al*., 2011; Padilla *et al*., 2013). However, it is unknown whether the production of superoxide production and pro-inflammatory proteins from PVAT is increased with aging, and whether superoxide signaling in PVAT contributes to large artery stiffening with age.

## Results

**Figure 1:**
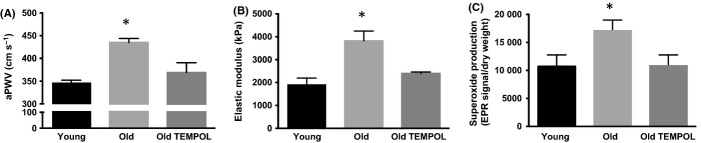
Large elastic artery stiffness and perivascular adipose tissue (PVAT) superoxide production with aging and in TEMPOL-treated old mice. (A) Aortic pulse wave velocity (aPWV), (B) *ex vivo* arterial stiffness, and (C) PVAT superoxide production in young, old, and old TEMPOL (1 mm)-treated mice (*N* = 4–6/group); Values are means ± S.E. *P < 0.05 vs. Young and Old TEMPOL.

Large elastic artery stiffness was greater in old compared with young control mice based on aortic pulse wave velocity (aPWV), the clinical gold standard measure (Sutton-Tyrrell *et al*., 2005; Mitchell *et al*., 2010) (Fig. 1A), and *ex vivo* intrinsic mechanical stiffness (Fig. 1B) (both, *P* < 0.05). Treatment with TEMPOL, a superoxide dismutase mimetic (Simonsen *et al*., 2009), reduced aortic stiffness in old mice to levels not different from young control animals (Fig. 1A,B). Aortic wall thickness, lumen diameter, and adventitial collagen I expression were greater in old compared with young control mice, and these differences were abolished with TEMPOL (Table S1 and Fig. S1) (all, *P* < 0.05). Arterial systolic, diastolic, and mean blood pressures were not different with aging or TEMPOL treatment (Table S1) as previously shown (Fleenor *et al*., 2012).

Superoxide production was increased in whole tissue samples of PVAT surrounding the thoracic aorta (Fig. 1C), and in adipocytes isolated from PVAT of old control compared with young control mice (Fig. S2) (both, *P* < 0.05). TEMPOL normalized aortic PVAT superoxide production in whole tissue samples from old mice to young control levels (Fig. 1C) (*P* < 0.05).

Because excessive superoxide production induces inflammation, we assessed the secretion of pro-inflammatory cytokines/chemokines in PVAT-conditioned media. The pro-inflammatory cytokines/chemokines GM-CSF, IL-6, CXCL1, CCL2/MCP-1, CXCL2 were greater in PVAT-conditioned media from old compared with young control mice (Fig. S3) (all, *P* < 0.05). C5/C5a and TIMP-1 were also detected in conditioned media from old mice, but not from young controls (Fig. S3).

**Figure 2:**
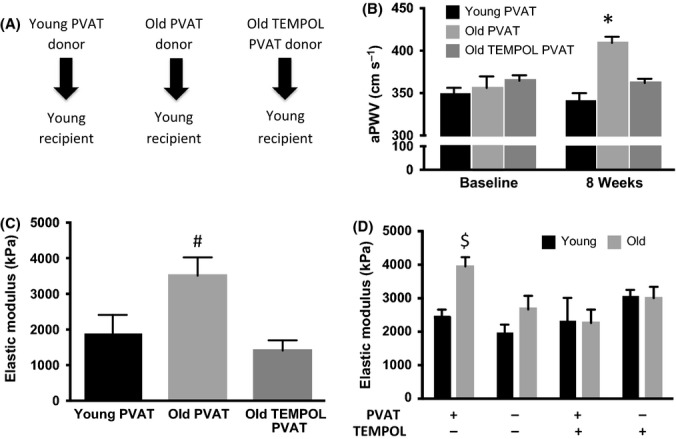
Effects of perivascular adipose tissue (PVAT) on large elastic artery stiffness. (A) Thoracic PVAT was removed from donor mice and transplanted into young recipient for 8 weeks. (B) Aortic pulse wave velocity (aPWV) and (C) *ex vivo* arterial stiffness in young recipient mice receiving PVAT from young, old, and old TEMPOL-treated donors after 8 weeks. (D) Arterial stiffness in aortic segments cultured for 72 h with (+) or without (−) perivascular adipose tissue from additional young and old control mice in the presence (+) or absence (−) of TEMPOL (*N* = 4–6/group); Values are means ± S.E. *P < 0.05 vs. all; # *P* < 0.05 vs. Young PVAT and Old TEMPOL PVAT; $*P* < 0.05 vs. Young; Old PVAT (−), TEMPOL (−) and Old PVAT (+), TEMPOL (+).

Thoracic aorta PVAT was removed from young control, old control, and old TEMPOL-treated donors and transplanted directly onto the abdominal aorta of young recipient mice for 8 weeks (Fig 2A). Young recipient mice transplanted with PVAT from old animals had greater aortic stiffness as indicated by increased aPWV and *ex vivo* intrinsic mechanical stiffness compared with those transplanted with PVAT from young donors (Fig. 2B,C) (both, *P* < 0.05). TEMPOL treatment in old donors abolished the increases in aPWV and *ex vivo* intrinsic mechanical stiffness observed with transplantation into young recipient mice (Fig 2B,C) (both, *P* < 0.05).

Wall thickness and adventitial collagen I expression were greater in young recipient mice transplanted with PVAT from old control compared with young control PVAT donors (both, *P* < 0.05), an effect that was not observed with PVAT donated from TEMPOL-treated old animals (Table S2 and Fig. S4). Lumen diameter and arterial systolic, diastolic, and mean blood pressures were not significantly different between groups (Table S2).

To further determine the effects of PVAT on arterial stiffness, aortic segments from additional young and old control mice were cultured in the presence (+) or absence (-) of PVAT for 72 h. Intrinsic mechanical stiffness was greater in aortic segments from old (+) PVAT compared with all aortic segments from young control mice (Fig. 2D) (all, *P* < 0.05). Compared with aortic segments from old (−) PVAT, samples from old (+) PVAT had greater intrinsic stiffness (Fig. 2D) (*P* < 0.05). TEMPOL reversed the intrinsic mechanical properties in arterial segments (+) PVAT to levels similar to old (−) PVAT (Fig. 2D) (*P* < 0.05).

## Discussion

The present study provides the first evidence directly linking PVAT with large elastic artery stiffness. The effects of PVAT from old mice in the promotion of arterial stiffening were demonstrated *in vivo* using a fat transplant model and in an *in vitro* tissue culture model. Importantly, superoxide production in PVAT from older animals was shown to be greater, and this was normalized with TEMPOL treatment, which, in turn, reversed PVAT-mediated arterial stiffening. The age-related increase in PVAT superoxide oxide production was associated with increased cytokine and chemokine secretion, indicating superoxide signaling may promote inflammation in PVAT. We also confirm the superoxide-lowering effect of TEMPOL to improve arterial function in old mice, suggesting excessive superoxide signaling is an important process in arterial stiffening (Fleenor *et al*., 2012). Arterial blood pressure can influence arterial stiffening; however, blood pressure was not different with aging or TEMPOL. Finally, our results suggest that superoxide signaling within PVAT may play an important role in the increase in aortic wall thickness and adventitial collagen I expression with aging.

Perivascular adipose tissue surrounding the thoracic aorta has been shown to resemble the phenotype of brown adipose tissue (Cannon & Nedergaard, 2004; Padilla *et al*., 2013). Assessing the phenotype, including inflammatory pathways, of thoracic aorta PVAT and whether the phenotype changes with transplantation is of interest and should be examined in future investigations.

In conclusion, our results provide the first evidence for aortic PVAT as a novel mechanism and potential therapeutic target in large elastic artery stiffening and increased CVD risk with aging.

## Supporting Information

**Data S1** Experimental procedures.

**Table S1** Arterial morphology and blood pressure in young, old and old TEMPOL-treated mice. **Table S2** Arterial morphology and blood pressure in young recipient mice after transplanted with PVAT from young, old or old TEMPOLtreated donors for 8 weeks. **Fig. S1** Adventitial collagen I expression in young, old and old TEMPOL-treated mice. **Fig. S2** Superoxide production in adipocytes isolated from PVAT of young and old mice. **Fig. S3** Cytokine secretion profile of cultured PVAT from young and old mice. **Fig. S4** Adventitial collagen I expression in young recipient mice transplanted with PVAT from young, old or old TEMPOL donors.

## References

[b1] Cannon B, Nedergaard J (2004). Brown adipose tissue: function and physiological significance. Physiol. Rev.

[b2] Findeisen HM, Pearson KJ, Gizard F, Zhao Y, Qing H, Jones KL, Cohn D, Heywood EB, De Cabo R, Bruemmer D (2011). Oxidative stress accumulates in adipose tissue during aging and inhibits adipogenesis. PLoS ONE.

[b3] Fleenor BS, Seals DR, Zigler ML, Sindler AL (2012). Superoxide-lowering therapy with TEMPOL reverses arterial dysfunction with aging in mice. Aging Cell.

[b4] Go AS, Mozaffarian D, Roger VL, Benjamin EJ, Berry JD, Borden WB, Bravata DM, Dai S, Ford ES, Fox CS, Franco S, Fullerton HJ, Gillespie C, Hailpern SM, Heit JA, Howard VJ, Huffman MD, Kissela BM, Kittner SJ, Lackland DT, Lichtman JH, Lisabeth LD, Magid D, Marcus GM, Marelli A, Matchar DB, McGuire DK, Mohler ER, Moy CS, Mussolino ME, Nichol G, Paynter NP, Schreiner PJ, Sorlie PD, Stein J, Turan TN, Virani SS, Wong ND, Woo D, Turner MB (2013). Heart disease and stroke statistics–2013 update: a report from the American Heart Association. Circulation.

[b5] Kim JH, Bugaj LJ, Oh YJ, Bivalacqua TJ, Ryoo S, Soucy KG, Santhanam L, Webb A, Camara A, Sikka G, Nyhan D, Shoukas AA, Ilies M, Christianson DW, Champion HC, Berkowitz DE (2009). Arginase inhibition restores NOS coupling and reverses endothelial dysfunction and vascular stiffness in old rats. J. Appl. Physiol.

[b6] Mitchell GF, Hwang S-J, Vasan RS, Larson MG, Pencina MJ, Hamburg NM, Vita JA, Levy D, Benjamin EJ (2010). Arterial stiffness and cardiovascular events: The Framingham Heart Study. Circulation.

[b7] Padilla J, Jenkins NT, Vieira-Potter VJ, Laughlin MH (2013). Divergent phenotype of rat thoracic and abdominal perivascular adipose tissues. Am. J. Physiol. Regul. Integr. Comp. Physiol.

[b8] Simonsen U, Christensen FH, Buus NH (2009). The effect of tempol on endothelium-dependent vasodilatation and blood pressure. Pharmacol. Ther.

[b9] Sindler AL, Fleenor BS, Calvert JW, Marshall KD, Zigler ML, Lefer DJ, Seals DR (2011). Nitrite supplementation reverses vascular endothelial dysfunction and large elastic artery stiffness with aging. Aging Cell.

[b10] Sutton-Tyrrell K, Najjar SS, Boudreau RM, Venkitachalam L, Kupelian V, Simonsick EM, Havlik R, Lakatta EG, Spurgeon H, Kritchevsky S, Pahor M, Bauer D, Newman A (2005). Elevated aortic pulse wave velocity, a marker of arterial stiffness, predicts cardiovascular events in well-functioning older adults. Circulation.

