# Supplementary material for: Superoxide signaling in perivascular adipose tissue promotes age-related artery stiffness
Source: Aging Cell. 2014 Jan 21;13(3):576–8. doi: 10.1111/acel.12196 (PMC4326900; doi:10.1111/acel.12196)
Supplement: Supplementary file 1 — Data S1 Experimental procedures. [file acel0013-0576-sd1.docx]

**Supporting Information**

**Experimental Procedures**

**Animals.** Young (4-6 months) and old (26-28 months) male C57BL/6N mice were purchased from the aged rodent colonies maintained by the National Institute of Aging. Mice were housed at the University of Colorado at Boulder in an animal facility on a 12 h:12 h light-dark cycle with *ad libitum* access to water and normal rodent chow. All animals were acclimated for 2 weeks prior to study or TEMPOL treatment. A group of old mice were treated with TEMPOL supplemented (1mM) drinking water for 3 weeks as described previously (Fleenor *et al.* 2012a). All animal procedures were approved by the UCB Animal Care and Use Committee and conformed to the *Guide for the Care and Use of Laboratory Animals* (8^th^ edition, revised 2011).

**Perivascular adipose tissue (PVAT) transplant.** The adipose tissue transplant technique was modified as described by others (Ohman *et al.* 2008; Ohman *et al.* 2011). Due to the technical limitations of accessing and transplanting fat onto the descending thoracic aorta in mice, PVAT was applied to the abdominal aorta as an *in vivo* model to determine the effects of this fat depot on arterial stiffness. As such, thoracic aorta PVAT was removed from young, old and old TEMPOL donor mice and surgically transplanted adjacent to the aorta in the abdominal cavity of young recipient mice for 8 weeks. aPWV was assessed prior to surgery and after the 8 week treatment as described below. After euthanasia, segments of aorta were excised for intrinsic mechanical testing (see below).

**Aortic pulse wave velocity (aPWV).** aPWV was measured as described previously by our laboratory and others (Kim *et al.* 2009; Sindler *et al.* 2011; Fleenor *et al.* 2012a; Fleenor *et al.* 2012b; Fleenor *et al.* 2013). Briefly, anesthetized mice (2% isoflurane) were placed supine on a heating board with legs secured to ECG electrodes. Non-invasive aortic velocities were measured with Doppler probes (Indus Instruments) at the transverse aortic arch and abdominal aorta. Pre-ejection times were calculated for both sites. To calculate aortic pulse wave velocity, the distance between the transverse and abdominal probes was divided by the difference in the thoracic and abdominal pre-ejection times and is presented as centimeters/second.

**Arterial blood pressure.** Non-invasive systolic and diastolic blood pressures were assessed using the CODA tail-cuff system (Kent Scientific) (Daugherty *et al.* 2009; Fleenor *et al.* 2012a). Mice were placed in restrainers on a warm pad for ~15 minutes, followed by 5 acclimation cycles and 20 data collection cycles to record systolic and diastolic blood pressure. This procedure was performed on three consecutive days and the data averaged.

**Intrinsic mechanical testing.** Intrinsic aortic stiffness was determined as previously described (Humphrey 2002; Fleenor *et al.* 2012b). Descending thoracic aortic segments were cleaned of the surrounding adipose tissue and loaded onto pins into a pre-heated (37° C) calibrated wire myograph (DMT) chamber with calcium- and magnesium-free phosphate buffered saline. Segments were incrementally stretched ~10% every 3 minutes with force recorded. Stress-strain curves were generated for the calculation of elastic modulus as described previously (Fleenor *et al.* 2012b).

**Aortic tissue culture.** Additional aortic segments from young and old control mice were cultured at 37° C in DMEM media for 72 hours either in the presence or absence of PVAT and/or TEMPOL (100 μM). The culture media was changed daily. After 72 hours of culture PVAT was removed from all arterial segments and the aorta underwent intrinsic mechanical testing.

**Perivascular adipose tissue superoxide production.** Superoxide production in thoracic aorta PVAT was assessed by electron paramagnetic resonance (EPR) spectrometry (Sindler *et al.* 2011; Fleenor *et al.* 2012a; Fleenor *et al.* 2013). Segments of PVAT or adipocytes isolated from PVAT were incubated for 60 min at 37º C in 200 μl of Krebs-HEPES buffer containing 0.55 mmol/L 1-hydroxy-3-methoxycarbonyl-2,2,5,5-tetramethylpyrrolidine (Alexis Biochemicals). Samples were analyzed immediately on an MS300 X-band EPR spectrometer (Magnettech, Berlin, Germany). Following the EPR measures, PVAT was placed in a 37° C incubator for 24 hours to dry the tissue. PVAT-derived adipocytes were isolated as described previously (Rodbell 1964; Police *et al.* 2009). Briefly, PVAT was minced and incubated in a collagenase (1mg/ml) solution for 1 hour at 37°C in a shaking water bath. Using a 100-micron filter the slurry was filtered into a 50mL conical tube, which was subsequently centrifuged at 500g for 10 minutes at 4°C. The adipocyte phase was used to assess superoxide production. The EPR signal was normalized to PVAT dry weight or cell number for the isolated cell preparation.

**Immunohistochemistry.** Collagen I staining was performed as described previously (Fleenor *et al.* 2012a; Fleenor *et al.* 2012b; Fleenor *et al.* 2013). Briefly, 7μm sections of aorta were stained using the Dako EnVision+System-HRP-DAB kit (Dako) as recommended by the manufacturer in a single batch. The collagen I primary antibody (1:4000, Milliipore) was incubated at 4°C for 1 hour. A 30-minute exposure to a labeled polymer secondary was followed by a 2-minute application of diaminobenzidine (DAB) to visualize the staining. Slides were dehydrated with increasing concentrations of ethanol (50-100%), cleared in xylenes and cover-slipped. Digital images were acquired with a Nikon Eclipse 80i microscope and densitometry analysis of the adventitial layer was performed with ImageJ software. Data are expressed relative to the young group.

**Cytokine and chemokine array on PVAT conditioned media.** Descending thoracic aorta PVAT was removed from young and old control mice that was cultured in DMEM (containing antibiotics only) at a concentration of 20mg fat per 1mL media for 24 hours. The conditioned media was assessed for 40 different cytokines/chemokines with the Proteome Profiler, Mouse Cytokine Array Panel A (R&D Systems) per the manufacturer instructions using 250μL of conditioned media for each sample. Membranes with conditioned media were incubated overnight at 4°C, washed, treated with streptavidin-HRP for 30-minutes and washed a second time. Chemiluminescence was used to visualize the expression. Duplicates for each cytokine/chemokine were analyzed with densitometry analysis and averaged. A t-test was used to determine significance between young and old for each cytokine/chemokine.

**Statistics.** The data are presented as mean ± SEM, and were analyzed with SPSS 20.0 software. A 1-way ANOVA was used to analyze aortic pulse wave velocity, intrinsic mechanical properties, superoxide production and immunohistochemistry data, and a repeated measure 1-way ANOVA was used to analyze the fat transplant aortic pulse wave velocity data. Tissue culture studies were analyzed with a 3-way ANOVA. A t-test was used for the isolated adipocyte superoxide production and the cytokine array data. Posthoc analyses were performed where appropriate. Significance was set a P<0.05.

**References**

Daugherty A, Rateri D, Hong L, Balakrishnan A (2009). Measuring blood pressure in mice using volume pressure recording, a tail-cuff method. *Journal of Visualized Experiments*. **15**, 1291.

Fleenor BS, Seals DR, Zigler ML, Sindler AL (2012a). Superoxide-lowering therapy with TEMPOL reverses arterial dysfunction with aging in mice. *Aging cell*. **11**, 269-276.

Fleenor BS, Sindler AL, Eng JS, Nair DP, Dodson RB, Seals DR (2012b). Sodium nitrite de-stiffening of large elastic arteries with aging: Role of normalization of advanced glycation end-products. *Experimental gerontology*. **47**, 588-594.

Fleenor BS, Sindler AL, Marvi NK, Howell KL, Zigler ML, Yoshizawa M, Seals DR (2013). Curcumin amerliorates arterial dysfunction and oxidative stress with aging. *Experimental gerontology*. **48**, 269-276.

Humphrey JD (2002). Cardiovascular solid mechanics: cells, tissues, and organs: Springer.

Kim JH, Bugaj LJ, Oh YJ, Bivalacqua TJ, Ryoo S, Soucy KG, Santhanam L, Webb A, Camara A, Sikka G, Nyhan D, Shoukas AA, Ilies M, Christianson DW, Champion HC, Berkowitz DE (2009). Arginase inhibition restores NOS coupling and reverses endothelial dysfunction and vascular stiffness in old rats. *J Appl Physiol*. **107**, 1249-1257.

Ohman MK, Luo W, Wang H, Guo C, Abdallah W, Russo HM, Eitzman DT (2011). Perivascular visceral adipose tissue induces atherosclerosis in apolipoprotein E deficient mice. *Atherosclerosis*.

Ohman MK, Shen Y, Obimba CI, Wright AP, Warnock M, Lawrence DA, Eitzman DT (2008). Visceral Adipose Tissue Inflammation Accelerates Atherosclerosis in Apolipoprotein E Deficient Mice. *Circulation*. **117**, 798-805.

Police SB, Thatcher SE, Charnigo R, Daugherty A, Cassis LA (2009). Obesity Promotes Inflammation in Periaortic Adipose Tissue and Angiotensin II-Induced Abdominal Aortic Aneurysm Formation. *Arteriosclerosis, thrombosis, and vascular biology*. **29**, 1458-1464.

Rodbell M (1964). Metabolism of Isolated Fat Cells. I. Effects of Hormones on Glucose Metabolism and Lipolysis. *The Journal of biological chemistry*. **239**, 375-380.

Sindler AL, Fleenor BS, Calvert JW, Marshall KD, Zigler ML, Lefer DJ, Seals DR (2011). Nitrite supplementation reverses vascular endothelial dysfunction and large elastic artery stiffness with aging. *Aging cell*. **10**, 429-437.
