# Supplementary material for: Superoxide signaling in perivascular adipose tissue promotes age-related artery stiffness
Source: Aging Cell. 2014 Jan 21;13(3):576–8. doi: 10.1111/acel.12196 (PMC4326900; doi:10.1111/acel.12196)

Supplemental Table I. Arterial morphology and blood pressure in young, old and old TEMPOL-treated mice.

	Young	Old	Old TEMPOL
**Wall Thickness (μM)**	61 ± 4	94 ± 13 *	77 ± 3
**Lumen Diameter (μM)**	748 ± 57	925 ± 91*	760 ± 18
**Arterial Blood Pressure (mmHg)**			
**Systolic**	105 ± 4	106 ± 5	103 ± 5
**Diastolic**	76 ± 4	74 ± 3	71 ± 4
**Mean**	85 ± 4	84 ± 4	82 ± 4

Values are mean ± S.E. * P < 0.05 vs. Young and Old TEMPOL

Supplemental Table II. Arterial morphology and blood pressure in young recipient mice after transplanted with PVAT from young, old or old TEMPOL-treated donors for 8 weeks.

	Young PVAT	Old PVAT	Old TEMPOL PVAT
**Wall Thickness (μM)**	62 ± 3	82 ± 3 *	68 ± 4
**Lumen Diameter (μM)**	648 ± 19	670 ± 28	668 ± 49
**Arterial Blood Pressure (mmHg)**			
**Systolic**	109 ± 5	111 ± 2	110 ± 7
**Diastolic**	75 ± 5	82 ± 2	82 ± 7
**Mean**	86 ± 5	91 ± 2	92 ± 8

Values are mean ± S.E. * P < 0.05 vs. Young PVAT and Old TEMPOL PVAT

Supplemental Figure I. Adventitial collagen I expression in young, old and old TEMPOL-treated mice. (A) Representative immunohistochemistry images of collagen I staining and (B) quantification of adventitial collagen in aortic sections from young, old and old TEMPOL (1mM) treated mice (N=3-4/group). Arrows denote the medial-adventitial border; bar = 100µm; Values are means ± S.E. * P < 0.05 vs. Young and Old TEMPOL

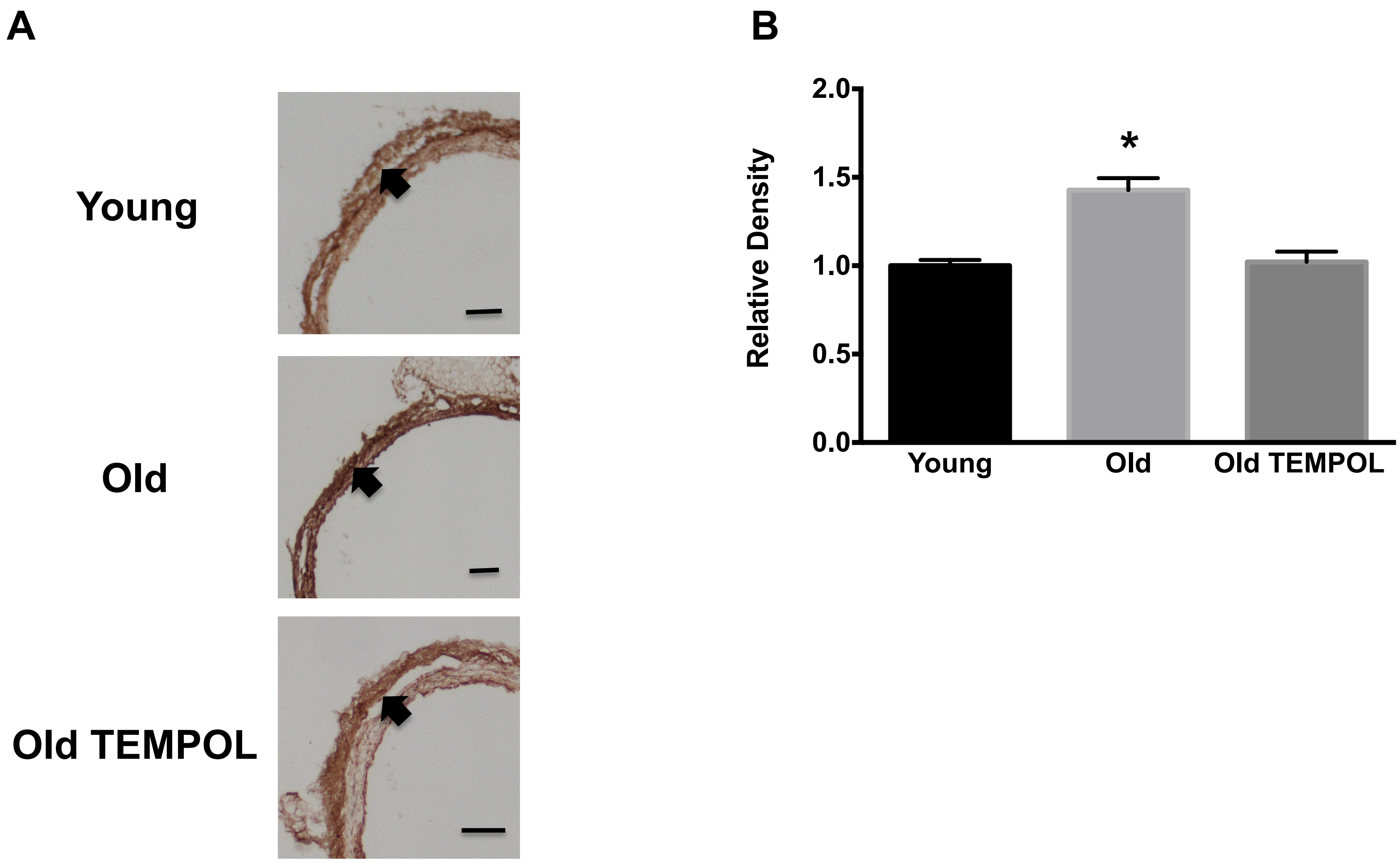

Supplemental Figure II. Superoxide production in adipocytes isolated from PVAT of young and old mice. PVAT-derived adipocytes isolated from young and old mice (N=3-5/group). Values are means ± S.E. * P < 0.05 vs. Young

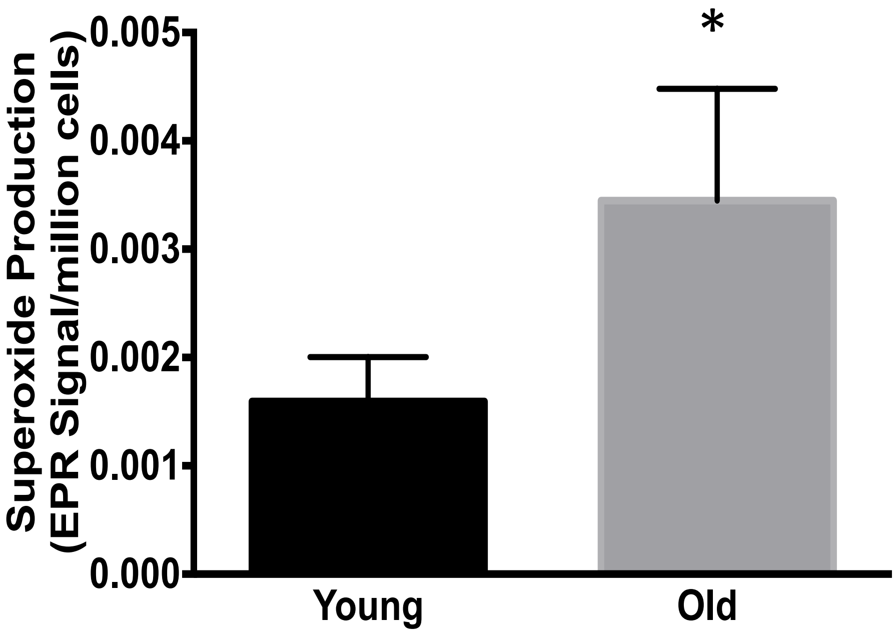

Supplemental Figure III. Cytokine secretion profile of cultured PVAT from young and old mice. (A) Cytokine array of secreted factors of cultured PVAT and (B) quantification from young and old mice. (C) Array coordinates for cytokine identification. n.d.: not detectable; N=3/group; Values are means ± S.E. * P < 0.05 vs. Young

A

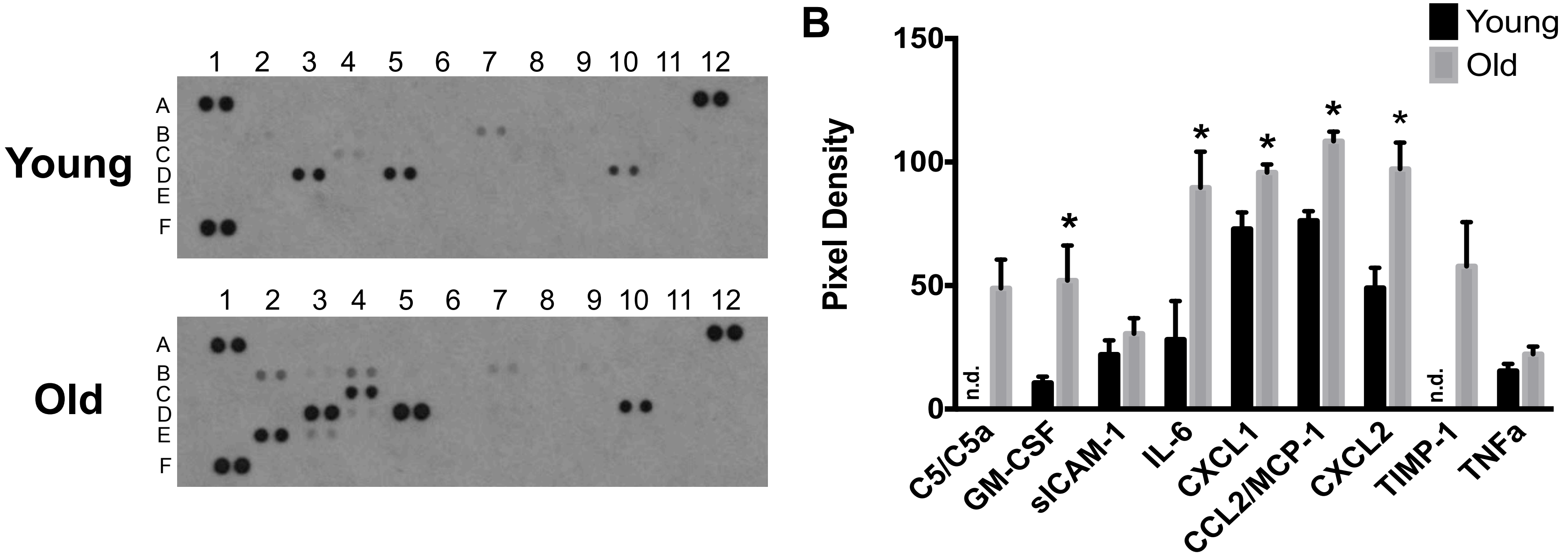

C

	1	2	3	4	5	6	7	8	9	10	11	12
A	Positive Control											Positive Control
B	CXCL13	C5/C5a	G-CSF	GM-CSF	CCL1	Eotaxin	sICAM-1	IFN-γ	IL-1α	IL-1β	IL-1ra	IL-2
C	IL-3	IL-4	IL-5	IL-6	IL-7	IL-10	IL-13	IL-12 p70	IL-16	IL-17	IL-23	IL-27
D	CXCL10	CXCL11	CXCL1	M-CSF	CCL2/ MCP-1	CCL12	CXCL9	CCL3	CCL4	CXCL2	CCL5	CXCL12
E	CCL17	TIMP-1	TNFα	TREM-1								
F	Positive Control											Negative Control

Supplemental Figure IV. Adventitial collagen I expression in young recipient mice transplanted with PVAT from young, old or old TEMPOL donors. (A) Representative immunohistochemistry images of collagen I staining and (B) quantification of adventitial collagen in aortic sections of young recipient mice after transplanted with PVAT from young, old and old TEMPOL (1mM) treated donor mice (N=3-5/group) for 8 weeks. Arrows denote the medial-adventitial border; bar = 100µm; Values are means ± S.E. * P < 0.05 vs. Young and Old TEMPOL

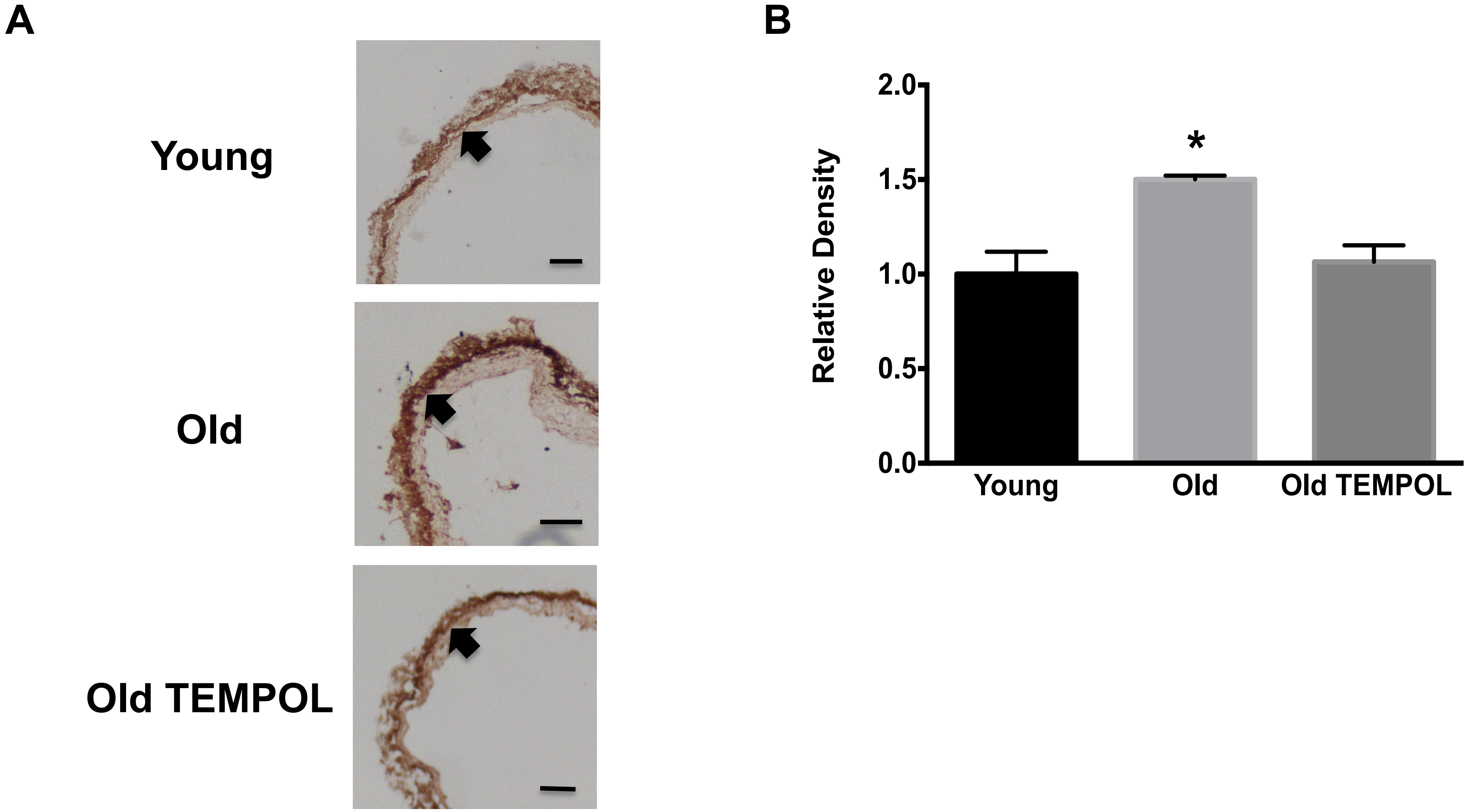

Supplement: Supplementary file 2 — Table S1 Arterial morphology and blood pressure in young, old and old TEMPOL-treated mice. Table S2 Arterial morphology and blood pressure in young recipient mice after transplanted with PVAT from young, old or old TEMPOLtreated donors for 8 weeks. Fig. S1 Adventitial collagen I expression in young, old and old TEMPOL-treated mice. Fig. S2 Superoxide production in adipocytes isolated from PVAT of young and old mice. Fig. S3 Cytokine secretion profile of cultured PVAT from young and old mice. Fig. S4 Adventitial collagen I expression in young recipient mice transplanted with PVAT from young, old or old TEMPOL donors. [file acel0013-0576-sd2.pdf]
